# Supplementary material for: Low QRS Voltage in Limb Leads Indicates Accompanying Precordial Voltage Attenuation Resulting in Underestimation of Left Ventricular Hypertrophy
Source: Int J Environ Res Public Health. 2021 Dec 7;18(24):12867. doi: 10.3390/ijerph182412867 (PMC8700898; doi:10.3390/ijerph182412867)
Supplement: Supplementary file 1 [file ijerph-18-12867-s001.zip › ijerph-1473122-supplementary.pdf]

Supplementary Table S1. Comparison of baseline characteristics between patients with LQRSV in limb leads and control group without LQRSV

	LQRSV in limb leads (n = 292)	Control (n = 194)	P value
Age (year)	71.3 ± 6.7	71.5 ± 6.1	0.78
Male	177 (60.6)	98 (50.5)	0.03
Height (cm)	160.7 ± 8.9	159.4 ± 8.8	0.20
Weight (kg)	62.0 ± 10.7	59.5 ± 10.1	0.01
BMI (kg/m^2)	23.9 ± 3.3	23.3 ± 3.0	0.03
Hypertension	150 (51.0)	98 (50.5)	0.85
Diabetes	78 (26.7)	50 (25.8)	0.82
Structural heart disease*	18 (6.2)	21 (10.8)	0.06
Ventricular dilatation	5 (1.7)	12 (6.2%)	0.01
Classic etiologies of LQRSV	40 (13.7)	7 (3.6)	<0.001
Infiltrative cardiomyopathy	0 (0.0)	0 (0.0)	0.999
Pericardial effusion, moderate to large	6 (2.0)	1 (0.5)	0.07
Pulmonary hyperinflation, emphysema, or obstructive lung disease	18 (6.2)	5 (2.6)	0.07
Pleural effusion, large to massive	3 (1.0)	0 (0.0)	0.16
BMI ≥ 30 kg/m^2	14 (4.8)	2 (1.0)	0.02

Values are presented as the mean ± SD or number (percentage). *Structural heart disease included left ventricular systolic dysfunction, valvular heart disease, ischemic or non-ischemic cardiomyopathy other than infiltrative cardiomyopathy. BMI, body mass index; LQRSV, low QRS voltage.
